# Chemical Investigation of Saponins in Different Parts of *Panax notoginseng* by Pressurized Liquid Extraction and Liquid Chromatography-Electrospray Ionization-Tandem Mass Spectrometry

**DOI:** 10.3390/molecules17055836

**Published:** 2012-05-16

**Authors:** Jian-Bo Wan, Qing-Wen Zhang, Si-Jia Hong, Peng Li, Shao-Ping Li, Yi-Tao Wang

**Affiliations:** 1 State Key Laboratory of Quality Research in Chinese Medicine (University of Macau), Macao, China; 2 Institute of Chinese Medical Sciences, University of Macau, Macao, China

**Keywords:** different parts of *Panax notoginseng*, saponin, pressurized liquid extraction, liquid chromatography-mass spectrometry

## Abstract

A pressurized liquid extraction (PLE) and high performance liquid chromatography-electrospray ionization tandem mass spectrometry (HPLC-ESI-MS/MS) method was developed for the qualitative determination of saponins in different parts of *P. notoginseng*, including rhizome, root, fibre root, seed, stem, leaf and flower. The samples were extracted using PLE. The analysis was achieved on a Zorbax SB-C18 column with gradient elution of acetonitrile and 8 mM aqueous ammonium acetate as mobile phase. The mass spectrometer was operated in the negative ion mode using the electrospray ionization, and a collision induced dissociation (CID) experiment was also carried out to aid the identification of compounds. Forty one saponins were identified in different parts of *P. notoginseng* according to the fragmentation patterns and literature reports, among them, 21 saponins were confirmed by comparing the retention time and ESI-MS data with those of standard compounds. The results showed that the chemical characteristics were obviously diverse in different parts of *P. notoginseng*, which is helpful for pharmacological evaluation and quality control of *P. notoginseng.*

## 1. Introduction

Root of *Panax notoginseng* (Burk.) F.H. Chen, also called Sanqi or Sanchi, is a highly valued Chinese medicinal herb. It has been used as a tonic and haemostatic in China for more than 400 years [1]. Modern pharmacological studies have revealed that *P. notoginseng* and its components exert various effects on immune system, cardiocerebral vascular system, central nervous system, endocrine system and inflammation, *etc.* [2,3,4]. Drammarane triterpenoid saponins with 20(*S*)-protopanaxatriol and 20(*S*)-protopanaxadiol aglycons have been considered the main active components of *P. notoginseng* [2,5]. Actually, the biological activities of saponins were related with their structures [6,7], ginsenoside Rg_1_, which has a protopanaxatriol moiety, and ginsenoside Rb_1_ with protopanaxadiol as aglycon, exhibited opposite pharmacological activity [6]. In our previous study [8], the chemical characteristics of different parts of *P. notoginseng* determined using HPLC-ELSD was described, and the results showed that the content and type of investigated saponins in different parts of *P. notoginseng* were distinctly diverse. Furthermore, due to detector limitations, many peaks in their profiles remained unknown. Therefore, further chemical investigation of different parts of *P. notoginseng* is necessary and very important for ensuring the safety, efficacy and their quality.

Several methods, including HPTLC [9], HPLC-UV [8], HPLC-ELSD [8,10,11], and UPLC-UV [12], have been developed by our lab for simultaneous determination of various saponins in the root of *P. notoginseng*. However, these methods suffer from insufficient sensitivity and poor identification capability for unknown peaks. Chromatography coupled with mass spectrometry is a powerful analytical tool for fast structural elucidation of complex mixtures. LC-MS has been applied to identify saponins in the roots of *P. notoginseng* [13,14,15,16], *P. ginseng* [17,18,19] and *P. quinquefolium* [20,21,22]. However, to date, the systematic HPLC-MS study of different parts of *P. notoginseng* has not been addressed. 

**Figure 1:**
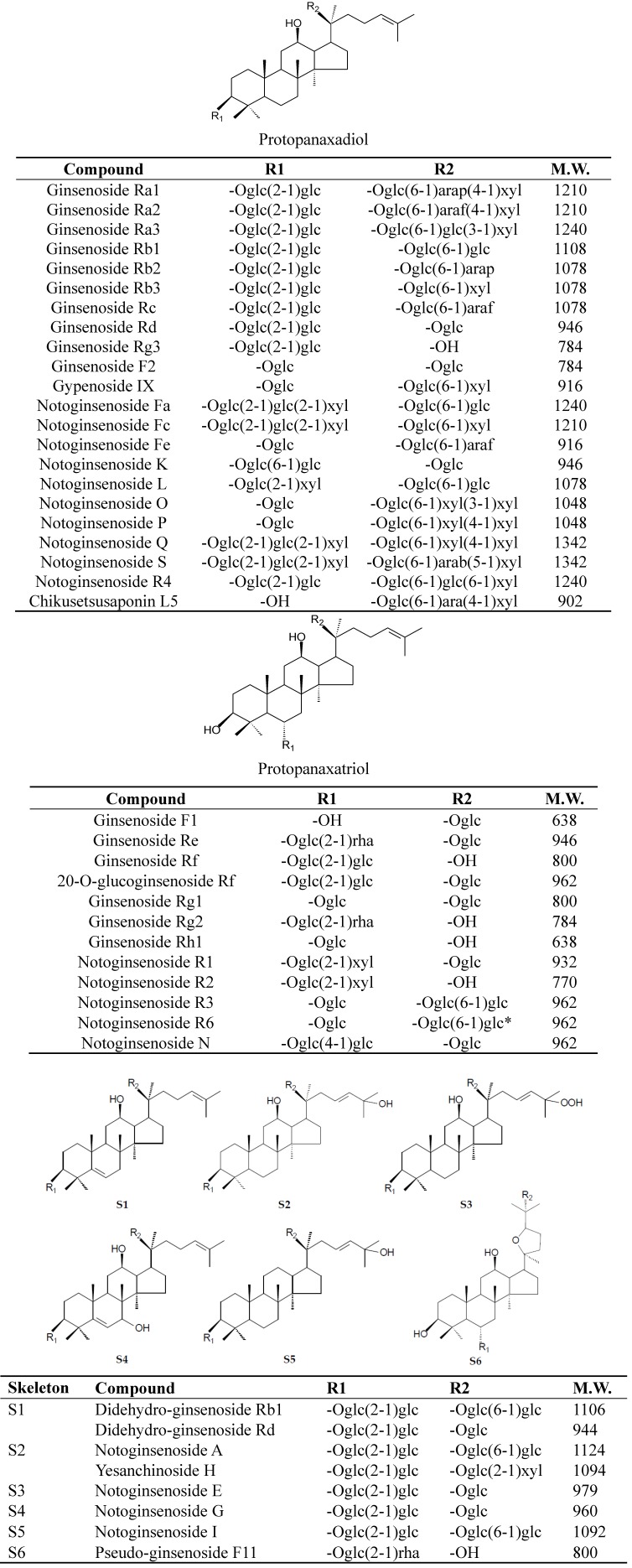
Chemical structures and molecular weight of saponins identified in different parts of *P. notoginseng*. Glc, β-D-glucopyranosyl; Rha, α-L-rhamnopyranosyl; Arap, α-L-arabinopyranosyl; Araf, α-L-arabinofuranosyl; Xyl, β-D-xylopyranosyl; Glc*, α-D-glucopyranosyl; M.W., molecular weight.

In the present study, a PLE and HPLC-ESI-MS/MS method was developed for separation and identification of saponins in different parts of *P. notogin*seng, including rhizome, root, fibre root, seed, stem, leaf and flower. Structural information of saponins was obtained by collision-induced dissociation (CID). Using the developed method, 41 saponins (Figure 1) were identified in different parts of *P. notoginseng* according to their fragmentation patterns and literatures; among them, 21 saponins were confirmed by comparing their retention times and ESI-MS data with those of standard compounds. Furthermore, the chemical characteristics of different parts of *P. notoginseng* were further compared.

## 2. Results and Discussion

### 2.1. Optimization of HPLC-ESI-MS/MS Conditions

Optimization of MS parameters is a necessary step to achieve maximum signal for the protonated molecular ions. In general, ion sensitivity for the ginsenosides in negative ion mode is greater than that in positive ion mode [17,23]. Our preliminary trials also confirmed that negative ion mode was more sensitive, and provided straightforward structural information about the saponins. The optimal range of capillary voltage is usually 2.0–3.5 kV for the negative ion study [24], and a capillary voltage of 3.0 kV was employed in this study. Eight mM aqueous ammonium acetate was used as mobile phase additive to improve analyte ionization prior to the HPLC-MS interface. However, the baseline drifted greatly at 203 nm using its gradient elution program. Therefore, water, instead of ammonium acetate solution, was used for HPLC-UV analysis. The other ESI-MS/MS parameters, including fragment amplification, compound stability, dry gas flow, dry temperature and nebulizer gas pressure, were optimized manually to obtain the maximum signal with chemical standards of ginsenosides Rb_1_ and Rg_1_, two main ingredients in the root of *P. notoginseng*, using a syringe pump (KD Scientific Model 100, Holliston, MA, USA) connected directly with the mass spectrometer.

### 2.2. HPLC-UV-MS Analysis of Reference Compounds

**Figure 2:**
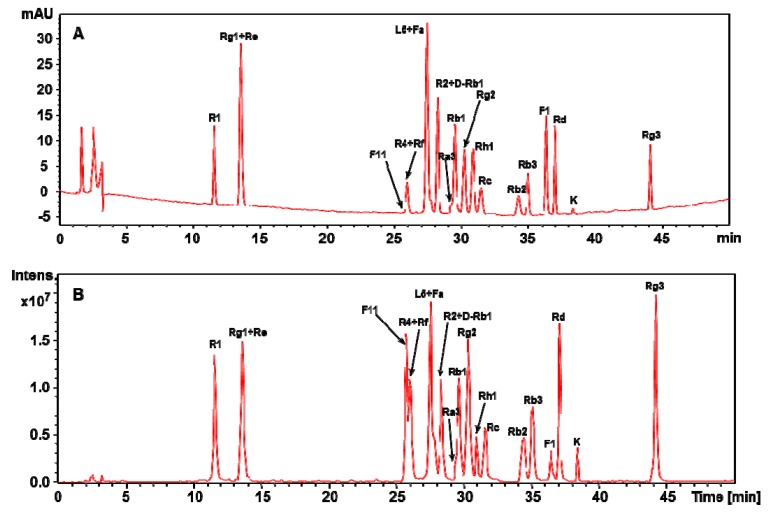
HPLC chromatograms of the mixed standards containing 21 saponins detected by (**A**) UV and (**B**) MS. D-Rb1, Didehydro-ginsenoside Rb_1_.

Under the optimized conditions, 21 reference compounds were analyzed by HPLC-UV-MS. The total ion chromatogram (TIC) of mixed standards was in good agreement with the HPLC-UV chromatogram (Figure 2). The majority of reference compounds were well-separated in 50 min. Pseudo-ginsenoside F_11_, an ocotillol type triterpene, lacked suitable chromophore and showed very poor UV absorption, so its corresponding peak was hardly detected at 203 nm (Figure 2A). However, it was readily detected using mass spectrometry, a universal, non-specific detector (Figure 2B). 

**Figure 3:**
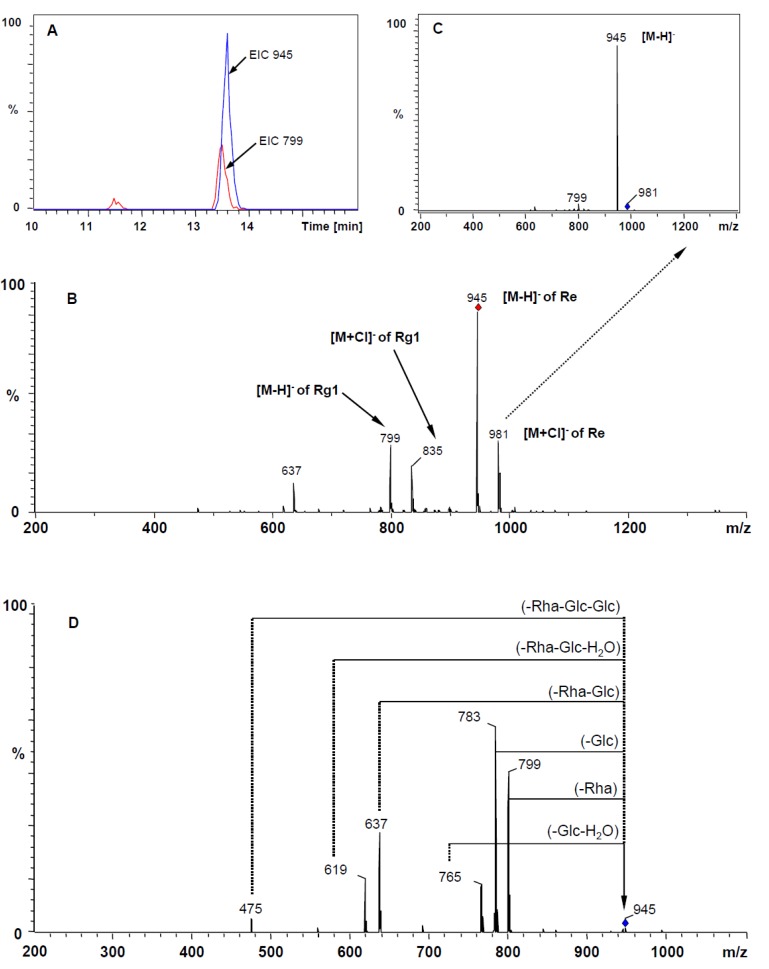
Mass spectra of ginsenoside Rg_1_ and Re in negative mode. (**A**) EIC of *m/z* 945 and 799; (**B**) HPLC-ESI-MS spectra; (**C**) the CID spectra of adduct ion [M+Cl]^−^ of ginsenoside Re at *m/z* 981; (**D**) the CID spectra of parent ion [M−H]^−^ of ginsenoside Re at *m/z* 945 (The deprotonated ion is indicated by a vertical arrow).

Although several pairs of saponins, including ginsenoside Rg_1_ and Re, ginsenoside Rf and notoginsenoside R_4_, chikusetsusaponin L_5_ and notoginsenoside Fa, notoginsenoside R_2_ and didehydro- ginsenoside Rb_1_, and ginsenoside Ra_3_ and Rb_1_, have identical retention times (Figure 2A), simultaneous determination could be accomplished by reference to the different MS of their molecular ions. For example, ginsenosides Rg_1_ and Re were co-eluted at the retention time of 13.5 min and failed to be distinguished with the UV detector (Figure 2A), but they were easily discriminated using extracted ion chromatograms (EIC) of their deprotonated molecular ions at *m/z* 799 and *m/z* 945, respectively (Figure 3A).

In the negative ion mass spectra, the ionization of ginsenosides afforded intense deprotonated molecular ions [M−H]^−^ together with their corresponding adduct ions [M+Cl]^−^, instead of the expected [M+AcO]^−^ ion. In general, the generation of adduct ions depends on the mobile phase modifier [17]. However, since chlorine-containing solvents were not employed during the whole HPLC-MS analysis, the presence of adduct ions [M+Cl]^−^ was considered to be generated from contamination of the ion source [25,26]. In the ESI-MS spectra of ginsenoside Rg_1_ and Re, their deprotonated molecular ions [M−H]^−^ (*m/z* 799 and 945, respectively) and adduct ions [M+Cl]^−^ (*m/z* 835 and 981, respectively) were observed with high abundance (Figure 3B). However, in the CID spectrum of the adduct ion at *m/z* 981 (Figure 3C) and *m/z* 835 (not shown), the [M−H]^−^ ions were produced from [M+Cl]^−^ ions corresponding to loss of one HCl unit, which could helped to confirm the quasi-molecular ions.

The negative MS/MS spectra were obtained by collision-induced dissociation (CID) from the deprotonated molecular ion [M−H]^−^ ions, and the mass spectra of product ion of [M−H]^−^ exhibited a fragmentation pattern corresponding to the successive loss of the glycosidic units till the formation of [aglycon-H]^−^ ions. In addition, according to the structural properties of saponins, 20(*S*)-protopanaxatriol- type saponins possessed an aglycon ion at *m/z* 475 which was visible for ginsenoside Rg_1_, Re, Rf, and Rg_2_, while 20(*S*)-protopanaxadiol-type saponins, including ginsenoside Rb_1_, Rc, Rb_2_, Rb_3_ and Rd, produced an aglycon ion at *m/z* 459. The amount and the type of sugar moieties were also determined from CID spectra in which a mass difference of 162 indicated the presence of a β-D-glucose, 132 the presence of a pentose [α-L-arabinose (pyranose or furanose) or β-D-xylose] and 146 the presence of an α-L-rhamnose.

In the CID spectra of ginsenoside Re, the parent ion at *m/z* 945 and six main fragment ions at *m/z* 799, 783, 765, 637, 619 and 475 were observed (Figure 3D). The mass differences between the parent ion and fragment ions *m/z* 799 and 783 were 146 and 162, which corresponded to loss of one rhamnose unit and one glucose unit, respectively. The simultaneous loss of two sugar units indicated the existence of two different terminal residues of the glycosidic moieties in its chemical structure. The fragment ions at *m/z* 637 corresponded to the loss of a disaccharide consisting of the one rhamnose and one glucose unit. The fragment ions *m/z* 765 and 619 were the daughter ions produced from *m/z* 945, corresponding to the loss of one or two sugar unit and one unit of H_2_O molecule. The fragment ion at *m/z* 475, corresponding to 20(*S*)-protopanaxatriol aglycon moiety, was the result of a subsequent loss of all linked glucose unit. Thus, the mass spectra of the selected ginsenoside was characterized by the observation of the two main types of fragment ions, the ions produced from the consecutive loss of saccharide unit and the ions resulting from loss of the H_2_O molecule. According to the information of ESI-MS and MS/MS, the saponins were easily identified by mass spectrometry. 

**Figure 4:**
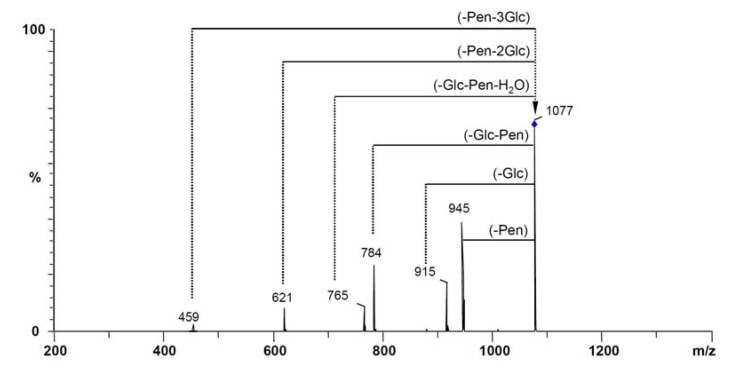
The CID spectra of parent ion [M−H]^−^ with *m/z* 1077. The deprotonated ion is indicated by a vertical arrow. Pen = pentose [arabinose or xylose].

However, the MS-based structural analysis faced difficulties in some cases. Ginsenoside Rc, Rb_2_ and Rb_3_, belonging to the (20*S*)-protopanaxadiol-type saponins, have the same molecular weight and fragment ions. In their parent ion CID spectrum (Figure 4), the four mass intervals of 162, 132, 162 and 162 were also observed, which indicated the presence of three glucose unit and one pentose unit. However, the pentose unit could be xylose, arabinose (pyranose) and arabinose (furanose), which are present in ginsenosides Rc, Rb_2_ and Rb_3_ respectively. These three isomers could be distinguished by their retention times, 31.5 min, 34.3 min and 35.0 min for ginsenoside Rc, Rb_2_ and Rb_3_ respectively, in the total ion chromatogram (TIC). 

Therefore, according to the TIC (retention time), ESI-MS (molecular weight) and MS/MS (fragment ion) information, the chromatographic behaviors and MS spectral of standards were characterized, which were the basis for identifying the ingredients in different parts of *P. notoginseng*.

### 2.3. HPLC-UV-MS Analysis of Different Parts of *P. notoginseng*

**Figure 5:**
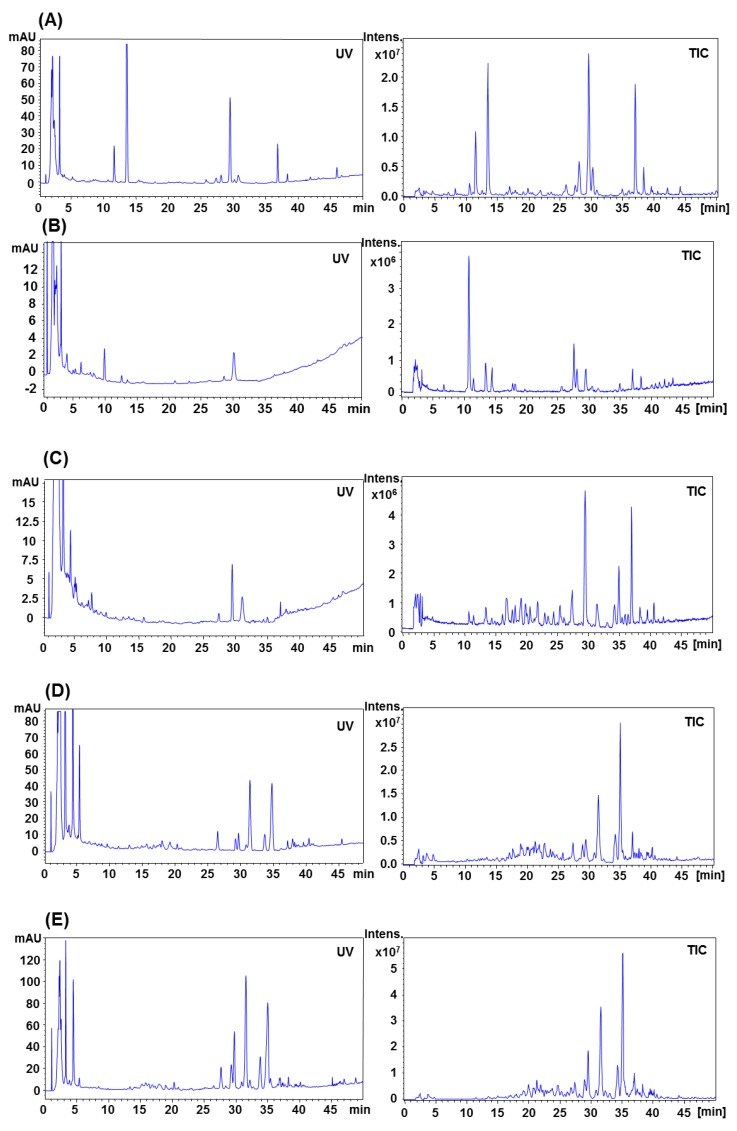
Representative HPLC-UV and MS chromatograms of different parts of *P. notoginseng*. (**A**) underground parts, (**B**) seed, (**C**) stem, (**D**) leaf and (**E**) flower, of *P. notoginseng*. UV, detected at the 203 nm; TIC, detected in negative ionization mode.

Figure 5 shows representative UV and TIC chromatograms of different parts of *P. notoginseng*, which were acquired in separate runs using mobile phases of water-acetonitrile and 8 mM aqueous ammonium acetate-acetonitrile, respectively. A total of 41 saponins were separated and identified within 50 min, their structures were tentatively identified by careful studies of MS and MS/MS spectra and by comparison with literature data. The peak identification of notoginsenoside R_1_ (5), ginsenoside Rg_1_ (7), Re (8), notoginsenoside R_4_ (13), ginsenoside Rf (14), chikusetsusaponin L_5_ (15), notoginsenoside Fa (16), notoginsenoside R_2_ (19), didehydroginsenoside Rb_1_ (21), ginsenoside Ra_3_ (24), Rb_1_ (25), Rg_2_ (26), Rh_1_ (27), Rc (28), Rb_2_ (30), Rb_3_ (31), F_1_ (34), Rd (35), notoginsenoside K (36) and ginsenoside Rg_3_ (41), were also confirmed by spiked injection of the authentic standards. 

The molecular mass could be clearly identified in their ESI-MS, and the type of ginsenoside and its sugar moiety could be further obtained using MS/MS. Therefore, those saponins that could not be identified from their retention times, due to lack of their chemical standards, could also be identified by their MS/MS spectral characteristics and by comparison with literature data. The MS data of characteristic peaks for identification are summarized in Table 1. 

**Figure 6:**
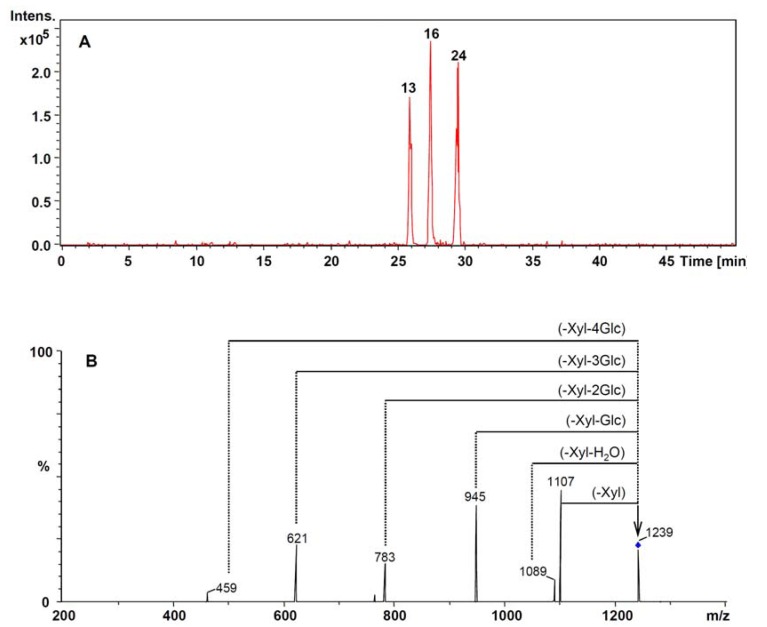
The extracted ion chromatogram of *m/z* 1,239 (**A**) and the CID spectra of parent ion [M−H]^−^ with *m/z* 1,239 (**B**) The mass-charge ratio of mass-selected ion is indicated by a vertical arrow.

Peaks 13, 16 and 24 exhibited molecular ions at *m/z* 1,239 [M−H]^–^ and 1,275 [M+CI]^–^ in the ESI-MS spectra of the underground parts of *P. notoginseng*. The retention times were 25.6 min, 27.3 min and 29.5 min, respectively, as shown in EIC of *m/z* 1,239 (Figure 6A). The mass differences between the parent ion and fragment ions indicated the existence of four glucose units and one xylose unit in their structures. In addition, the fragment ion at *m/z* 459 corresponding to the protopanaxadiol aglycon moiety was observed in the CIDs of their parent ions (Figure 6B). Hence, the three peaks were finally identified as notoginsenoside R_4_, ginsenoside Fa and Ra_3_, respectively, which was confirmed by spiking with their chemical standards.

**Table 1:** HPLC-ESI-MS and corresponding CID data of saponins in different parts of *P. notoginseng.*

No	Peak identification	RT(min)	MS ( *m/z*)		CID( *m/z*)	UG	a	b	c	d
			[M−H]^−^	[M+Cl]^−^						
1	Yesanchinoside H	7.2	1093	1129	961[M−H-Xyl]^−^, 931[M−H-Glc]^−^, 799[M−H-Xyl-Glc]^−^, 637[M−H-Xyl-2Glc]^−^, 475 [M−H-Xyl-2Glc]^−^	+	-	-	-	-
2	Notoginsenoside R_3_/R_6_/N/ 20-O-glucoginsenoside Rf	8.4	961	997	799[M−H-Glc]^−^, 637[M−H-2Glc]^−^, 475[M−H-3Glc]^−^	+	-	-	-	-
3	Notoginsenoside R_3_/R_6_/N/ 20-O-glucoginsenoside Rf	10.6	961	997	799[M−H-Glc]^−^, 637[M−H-2Glc]^−^, 475[M−H-3Glc]^−^	+	-	-	-	-
4	Notoginsenoside R_3_/R_6_/N/ 20-O-glucoginsenoside Rf	11.2	961	997	799[M−H-Glc]^−^, 637[M−H-2Glc]^−^, 475[M−H-3Glc]^−^	+	-	-	-	-
5	Notoinsenoside R_1_	11.5	931	967	799[M−H-Xyl]^−^, 769[M−H-Glc]^−^, 751[M−H-Glc-H_2_O]^−^, 637[M−H-Glc-Xyl]^−^, 619[M−H-Glc-Xyl-H_2_O]^−^, 475[M−H-Xyl-2Glc]^−^	+	+	+	+	+
6	Notoinsenoside R_1_ isomer	12.6	931	967	799[M−H-Xyl]^−^, 769[M−H-Glc]^−^, 637[M−H-Glc-Xyl]^−^, 475[M−H-Xyl-2Glc]^−^	+	-	-	-	-
7	Ginsenoside Rg_1_	13.6	799	835	637[M−H-Glc]^−^, 619[M−H-Glc-H_2_O]^−^, 475[M−H-2Glc]^−^	+	+	+	+	+
8	Ginsenoside Re	13.6	945	981	799[M−H-Rha]^−^, 783[M−H-Glc]^−^, 765.6[M−H-Glc-H_2_O]^−^, 637[M−H-Rha-Glc]^−^, 619[M−H-Rha-Glc-H_2_O]^−^, 475[M−H-Rha-2Glc]^−^	+	+	+	+	+
9	Notoginsenoside E	15.3	979	1015	817[M−H-Glc]^−^, 799[M−H-Glc-H_2_O]	+	-	-	-	-
10	Notoginsenoside A	15.5	1123	1159	-	+	-	-	-	-
11	Ginsenoside G	17.9	959	996	797[M-Glc], 635[M-2Glc]^−^, 473[M-3Glc]^−^	+	-	-	-	-
12	Notoginsenoside R_3_/R_6_/N/ 20-O-Glucoginsenoside Rf	21.9	961	998	799[M−H-Glc]^−^, 637[M−H-2Glc]^−^, 619[M−H-2Glc-H_2_O]^−^, 475[M−H-3Glc]^−^	+	-	-	-	-
13	Notoginsenoside R_4_	26.0	1239	1276	1107[M−H-pen]^−^, 945[M−H-xyl-Glc]^−^, 783[M−H-xyl-2Glc]^−^, 621[M−H-xyl-3Glc], 459[M−H-xyl-4Glc]^−^	+	-	-	-	-
14	Ginsenoside Rf	26.1	799	835	637[M−H-Glc]^−^, 619[M−H-Glc-H_2_O]^−^, 475[M−H-2Glc]^−^,	+	-	+	-	-
15	Chikusetsusaponin L_5_	27.2	901	937	769[M−H-Xyl]^−^, 637[M−H-Xyl-Ara]^−^, 475[M−H-Xyl-Ara-Glc]^−^	+	-	+	+	+
16	Notoginsenoside Fa	27.3	1239	1275	1107[M−H-Xyl]^−^, 1089[M−H-Xyl-H_2_O]^−^, 945[M−H-Xyl-Glc]^−^, 783[M−H-Xyl-2Glc]^−^, 621[M−H-Xyl-3Glc]^−^, 459[M−H-Xyl-4Glc]^−^	+	-	+	+	+
17	Notoinsenoside Q/S	27.7	1341	1377	1209[M−H-Xyl]^−^, 1192[M−H-Xyl-H_2_O]^−^, 1077[M−H-2Pen]^−^, 945[M−H-3Pen]^−^, 783[M−H-3Pen-Glc]^−^, 621[M−H-3Pen-2Glc]^−^, 459[M−H-3Pen-3Glc]^−^	-	-	-	-	+
18	Notoginsenoside I	27.7	1091	1127	929[M−H-Glc]^−^, 767[M−H-2Glc]^−^, 605[M−H-3Glc]^−^, 443[M−H-3Glc]^−^	+	-	-	-	-
19	Notoinsenoside R_2_	28.0	769	705	637[M−H-Xyl]^−^, 619[M−H-Xyl-H_2_O]^−^, 475[M−H-Xyl-Glc]^−^	+	-	-	-	-
20	Ginsenoside Fc/Ra_1_/Ra_2_	28.1	1209	1245	1077[M−H-Xyl]^−^, 1047[M−H-Glc]^−^, 1027[M−H-Glc-H_2_O]^−^, 945[M−H-Xyl-arab]^−^,915[M−H-Xyl-Glc]^−^, 897[M−H-Xyl-Glc-H_2_O]^−^, 783[M−H-Xyl-arab-Glc]^−^ 621[M−H-Xyl-arab-2Glc]^−^, 459[M−H-Xyl-arab-3Glc]^−^	-	-	-	-	+
21	Didehydroginsenoside Rb_1_	28.2	1105	1141	943[M−H-Glc]^−^, 781[M−H-2Glc]^−^, 619[M−H-3Glc]^−^, 457[M−H-4Glc]^−^	+	-	-	-	-
22	Ginsenoside Fc/Ra_1_/Ra_2_	28.9	1209	1245	1077[M−H-Xyl]^−^, 1059[M−H-Xyl-H_2_O]^−^, 1047[M−H-Glc]^−^, 945[M−H-Xyl-arab]^−^,915[M−H-Xyl-Glc]^−^, 783[M−H-Xyl-arab-Glc]^−^, 621[M−H-Xyl-arab-2Glc]^−^, 459[M−H-Xyl-arab-3Glc]^−^	-	-	+	+	+
23	Notoinsenoside Q/S	29.1	1341	1377	1209[M−H-Xyl]^−^, 1077[M−H-2Pen]^−^, 945[M−H-3Pen]^−^, 783[M−H-3Pen-Glc]^−^, 621[M−H-3Pen-2Glc]^−^, 459[M−H-3Pen-3Glc]^−^	-	-	-	-	+
24	Ginsenoside Ra_3_	29.5	1239	1275	1107[M−H-Xyl]^−^, 1089[M−H-Xyl-H_2_O]^−^, 945[M−H-Xyl-Glc]^−^, 783[M−H-Xyl-2Glc]^−^, 621[M−H-Xyl-3Glc]^−^, 459[M−H-Xyl-4Glc]^−^	+	-	-	-	-
25	Ginsenoside Rb_1_	29.6	1107	1143	945 [M−H-Glc]^−^, 783[M−H-2Glc]^−^, 621[M−H-3Glc]^−^, 459[M−H-4Glc]^−^	+	+	+	+	+
26	Ginsenoside Rg_2_	30.2	783	819	637[M−H-Rha]^−^, 619[M−H-Rha-H_2_O]^−^, 475[M−H-Rha-Glc]^−^	+	+	+	-	-
27	Ginsenoside Rh_1_	30.9	637	673	475[M−H-Glc]^−^	+	-	-	-	-
28	Ginsenoside Rc	31.5	1077	1113	945[M−H-Araf]^−^, 915[M−H-Glc]^−^, 783[M−H-Araf-Glc]^−^, 765[M−H-Araf-Glc-H_2_O]^−^, 621[M−H-Araf-2Glc]^−^, 603[M−H-Araf-2Glc-H_2_O]^−^, 459[M−H-Araf-3Glc]^−^	+	+	+	+	+
29	Ginsenoside Fc/Ra_1_/Ra_2_	31.6	1209	1245	1077[M−H-Xyl]^−^, 1047[M−H-Glc]^−^, 945[M−H-2Xyl/(-Xyl-Ara)]^−^, |783[M−H-(2Xyl-Glc)/(-Xyl-Ara-Glc)]^−^	+	-	-	-	-
30	Ginsenoside Rb_2_	34.3	1077	1113	945[M−H-Xyl]^−^, 915[M−H-Glc]^−^, 783[M−H-Araf-Glc]^−^, 765[M−H-Xyl-Glc-H_2_O]^−^, 621[M−H-Xyl-2Glc]^−^, 603[M−H-Xyl-2Glc-H_2_O]^−^, 459[M−H-Xyl-3Glc]^−^	+	+	+	+	+
31	Ginsenoside Rb_3_	35.0	1077	1113	945[M−H-Xyl]^−^, 915[M−H-Glc]^−^, 783[M−H-Araf-Glc]^−^, 765[M−H-Xyl-Glc-H_2_O]^−^, 621[M−H-Xyl-2Glc]^−^, 603[M−H-Xyl-2Glc-H_2_O]^−^, 459[M−H-Xyl-3Glc]^−^	+	+	+	+	+
32	Notoginsenoside L	35.5	1077	1113	945[M−H-Xyl]^−^, 915[M−H-Glc]^−^, 783[M−H-Xyl-Glc]^−^, 765[M−H-Xyl-Glc-H_2_O]^−^, 621[M−H-Xyl-2Glc]^−^, 603[M−H-Xyl-2Glc-H_2_O]^−^,459[M−H-Xyl-3Glc]^−^	+	+	+	+	+
33	Didehydroginsenoside Rd	35.7	943	979	781[M−H-Glc]^−^, 619[M−H-2Glc]^−^, 457[M−H-3Glc]^−^	+	-	-	-	-
34	Ginsenoside F_1_	36.3	637	673	475[M−H-Glc]^−^	+	-	-	-	-
35	Ginsenoside Rd	37.0	945	981	783[M−H-Glc]^−^, 765[M−H-Glc-H_2_O]^−^, 621[M−H-2Glc]^−^, 603[M−H-2Glc-H_2_O]^−^, 459[M−H-3Glc]^−^	+	+	+	+	+
36	Notoginsenoside K	38.3	945	981	783[M−H-Glc]^−^, 775[M−H-Glc-H_2_O]^−^, 621[M−H-2Glc]^−^, 459[M−H-3Glc]^−^	+	+	+	+	+
37	Notoginsenoside O/P	38.6	1047	1083	915[M−H-Xyl]^−^, 897[M−H-Xyl-H_2_O]^−^, 783[M−H-2Xyl]^−^, 621 [M−H-2Xyl-Glc]^−^,459[M−H-2Xyl-2Glc]^−^	-	-	-	+	+
38	Gypenoside IX/Notoginsenoside Fe/isomer	39.5	915	951	783[M-pen]^−^, 621[M-pen-glc]^−^, 459[M-pen-2glc]^−^	+	-	-	+	+
39	Gypenoside IX/ Notoginsenoside Fe/isomer	39.7	915	951	783[M-pen]^−^, 621[M-pen-glc]^−^, 459[M-pen-2glc]^−^	+	-	+	+	+
40	Ginsenoside F_2_	42.1	783	819	621[M−H-Glc]^−^, 603[M−H-Glc-H_2_O]^−^, 459[M−H-2Glc]^−^	+	-	-	-	-
41	Ginsenoside Rg_3_	44.1	783	819	a621[M−H-Glc]^−^, 603[M−H-Glc-H_2_O]^−^, 459[M−H-2Glc]^−^	+	-	+	+	+

UG, underground parts of *P. notoginseng* (including rhizome, root and fibre root); a, Seed; b, Stem; c, Leaf; d, Flower. -, Not detected; +, Detected.

Four peaks were observed to have molecular ions at *m/z* 961 [M−H]^−^ and *m/z* 997 [M+CI]^−^ in the ESI-MS of the underground parts of *P. notoginseng*. Their retention times were 8.4 min (2), 10.6 min (3), 11.2 min (4) and 21.9 min (12), respectively. The fragment ion at *m/z* 475 corresponding to the 20(*S*)-protopanaxatriol aglycon moiety was visible in CID spectra. Hence, the four peaks were tentatively identified as notoginsenoside R_3_, R_6_, N or 20-*O*-glucoginsenoside Rf, which possess the same molecular weight of 962 and belong to the 20(*S*)-protopanaxatriol type saponins [27,28]. Similarly, peaks 17 and 23 were identified as notoginsenoside Q or S in the leaf and flower of *P. notoginseng*, which were isomers with molecular weight of 1,342 and belonged to 20(*S*)-protopanaxadiol type saponins [28].

The pseudoginsenoside F_11_, an ocotillol-type saponin isolated from *P. quinquefolius*, was not detected in any part of *P. notoginseng*. Thus, pseudoginsenoside F_11_ could be a marker to distinguish *P. quinquefolius* from *Panax* genus plants [11]. Liu *et al* reported that the malonylginsenosides Rb_1_, Rd and Rg1 were identified in the root of *P. notoginseng* using HPLC-ESI-MS [29]. In this study, no malonylginsenoside was detected. The discrepancy between Liu’s report and our study may be caused by the low contents of these compounds and their thermally sensitive degradation in the PLE extraction utilized in the present study. Several minor saponins were detected in the underground parts and tentatively identified as yesanchinoside H (1), notoginsenosides E (10), A (11), G (13), I (22), L (36) and didehydroginsenoside Rd (33), by comparing their MS spectra with literature data [27,28,30,31,32]. 

The results showed that identical chemical ingredients presented in the underground parts of *P. notoginseng*, including root, fibre root and rhizome. However, the saponins were obviously different among the underground parts, seed, stem, leaf and flower. In total 36 peaks were identified in the underground parts of *P. notoginseng* (root, fibre root and rhizome), while 11, 17, 18 and 20 saponins were identified in the seed, stem, leaf and flower, respectively. Furthermore, the components of peaks 1–4, 6, 9–13, 18–19, 21, 24, 27, 29, 33, 34 and 40 were unique to the underground parts, notoinsenoside Q/S, ginsenoside Fc/Ra1/Ra2 were only found in flowers, and the notoginsenoside O/P were specifically contained in the leafs and flowers. 

## 3. Experimental

### 3.1. Chemicals, Standards and Samples

The samples of different parts from *P. notoginseng*, including rhizome, root, fibre root, seed, stem, leaf and flower, were derived from same batch of plants, obtained from Wenshan region, Yunnan Province, China. The peeled seed was used in this study. The botanical origins of materials were identified by Dr. Xiu-Ming Cui, Wenshan Prefecture Sanqi Research Institute, Yunnan Province. The voucher specimens were deposited at the Institute of Chinese Medical Sciences, University of Macau, Macao, China.

Notoginsenoside R_1_ was supplied by the National Institute for the Control of Pharmaceutical and Biological Products (Beijing, China); pseudo-ginsenoside F11, ginsenosides Rg_1_, Re, Rf, Rb_1_, Rg_2_, Rh_1_, Rc, Rb_2_, Rb_3_, Rd and Rg_3_ were purchased from International Laboratory (South San Francisco, CA, USA); Chikusetsusaponin L_5_, ginsenoside R_2_, F_1_, Ra_3_, notoginsenoside K, R_4_, Fa and didehydroginsenoside Rb_1_ were previously isolated and purified from the root of *P. notoginseng* by repeated silica gel, medium pressure liquid chromatography (MPLC) and preparative high performance liquid chromatography (prep-HPLC). Their structures were elucidated by comparison of their spectral data (MS, ^1^H-NMR and ^13^C-NMR) with corresponding references (Figure 1). Didehydroginsenoside Rb_1_ was a novel triterpene saponin we isolated from *P. notoginseng*, and its chemical structure was previously reported [32]. Their purities were determined to be higher than 97% by normalization of the peak areas detected by HPLC-UV. HPLC-grade methanol and acetonitrile were products of Merck (Darmstadt, Germany), and the deionized water was purified by a Milli-Q purification system (Millipore, Bedford, MA, USA). Ammonium acetate was purchased from Riedel-de Haën (Seelze, Germany).

### 3.2. Pressurized Liquid Extraction

Sample preparation was performed with a pressurized liquid extraction (PLE), which was operated on a Dionex ASE 200 system (Dionex Corp., Sunnyvale, CA, USA) under optimized conditions reported before [33]. In brief, dried sample powder (0.5 g) was placed in an 11 mL stainless steel extraction cell. The optimized conditions were: particle size, 0.3–0.45 mm; solvent, methanol; temperature, 150 °C; static time, 15 min; pressure, 6.895 × 10^3^ MPa; static cycles, 1, and number of extractions, 1. PLE extract was transferred into a 25 mL volumetric flask which was brought up to its volume with the same solvent and filtered through a 0.45 μm Econofilter (Agilent Technologies, Palo Alto, CA, USA) prior to injection into the HPLC system.

### 3.3. HPLC-UV Analysis

An Agilent 1100 series HPLC apparatus (Agilent Technologies), equipped with a vacuum degasser, quaternary gradient pump, autosampler and DAD detector, was used. A Zorbax SB-C18 column (250 mm × 4.6 mm, I.D., 5 μm) and a Zorbax SB-C18 guard column (12.5 mm × 4.6 mm I.D., 5 µm) were used. The mobile phase consisted of water (A) and acetonitrile (B) with the following gradient program: 0–3 min, 20–22% B; 3–25 min, 22–33% B; 25–30 min, remaining 33% B; 30–50 min, 33–70% B. The flow-rate was kept at 1.0 mL·min^−1^. The temperature was 25 °C. The detection wavelength was set at 203 nm and the sample injection volume was 10 µL.

### 3.4. HPLC-ESI-MS/MS Analysis

An LC-MSD trap VL mass spectrometer with electrospray ionization source (Agilent Technologies,) was coupled to the HPLC system described in Section 3.3. The chromatographic conditions for the HPLC-MS were the same as those used for the HPLC-UV, except that the water (A) of mobile phase was replaced by 8 mM aqueous ammonium acetate. The ESI-MS conditions were as follows: negative-ion mode; capillary voltage, 3.5 kV; dry gas N_2_, 12 L·min^−1^; temperature, 350 °C; pressure of nebulizer, 40 psi. The ESI-MS/MS was set with compounds stability of 100%, fragment amplification of 2.0 V and isolation width of 4. Scan range of both ESI-MS and ESI-MS/MS was fixed at *m/z* 200–1,400 U.

## 4. Conclusions

In conclusion, an HPLC-ESI-MS/MS and PLE method was developed for the extraction and qualitative determination of saponins in different parts of *P. notoginseng*, including the rhizome, root, fiber root, seed, stem, leaf and flower. A total 41 saponins were identified and the chemical profiles of the different parts were obviously diverse. The developed HPLC-ESI-MS/MS method provided a reliable means of distinguishing the saponins in different parts of *P. notoginseng*, which should be helpful for the pharmacological evaluation and quality control of *P. notoginseng*.
